# Assessing the Stiffness Perception of Acupressure Massage Beginning Learners: A Pilot Study

**DOI:** 10.3390/s21072472

**Published:** 2021-04-02

**Authors:** Kouki Doi, Saito Sakaguchi, Takahiro Nishimura, Hiroshi Fujimoto, Shuichi Ino

**Affiliations:** 1 Department of Information and Support, National Institute of Special Needs Education, Yokosuka 239-8585, Japan; 2 Graduate School of Human Sciences, Waseda University, Tokorozawa 359-1192, Japan; w.saito.c7@ruri.waseda.jp; 3 Center for Promoting Education for Persons with Developmental Disabilities, National Institute of Special Needs Education, Yokosuka 239-8585, Japan; nishimura@nise.go.jp; 4 Faculty of Human Sciences, Waseda University, Tokorozawa 359-1192, Japan; fujimoto@waseda.jp; 5 Human Informatics and Interaction Research Institute, National Institute of Advanced Industrial Science and Technology, Tsukuba 305-8566, Japan; s-ino@aist.go.jp

**Keywords:** assessment material, stiffness perception, acupressure massage, visual impairment, beginning learner

## Abstract

Visually impaired licensed therapists must have the ability to perceive stiffness through their fingertips in the school for the blind. The teachers strive to provide careful introductory education based on a quantitative assessment of new students’ basic stiffness perception. However, assessment materials to help teachers understand new students’ stiffness perception are lacking. This study aimed to develop suitable fundamental assessment materials that visually impaired licensed teachers could use to quantitatively assess the difference in the stiffness perception ability of beginning learners in the early stages of learning. They were asked to discriminate the presented materials one at a time, which consisted of thermoplastic elastomers with different degrees of stiffness. We used these materials to compare the beginning learners’ ability to perceive stiffness with that of teachers and found that teachers answered correctly at an overall significantly higher rate. Specifically, the teachers’ correct response rate (78.8%) for the stiffness perception of all presented stimuli was approximately 15% higher than the beginning learners’ correct response rate (64.2%). These results revealed areas of stiffness that are difficult for beginning learners to identify.

## 1. Introduction

Human beings use their five senses—sight, hearing, touch, taste, and smell—to recognize various stimuli in daily life [1]. Touch is sensed through cutaneous sensory receptors throughout the body [2]. The external stimuli that humans perceive through the skin include pressure, vibration, and temperature. However, external stimuli can also be perceived in muscles and joints [3]. Whereas human skin has sensory receptors on its surface [4], deeper parts of the human body use deep sensory receptors [5]. Such receptors acquire a vast amount and wide range of information, including the degree of body stiffness [6]. Study of the human sense of touch advances fields such as haptics, robotics, and medical telepresence [7,8]. Robotic research also includes remote and invasive measurements of stiffness for haptic information transmission [9,10]. 

Studies on stiffness perception have reported that depression displacement into an object [6,11,12], skin deformation [6,13,14], pushing force [15], and pushing speed [16] influence the human finger’s ability to perceive stiffness. However, these studies used a limited range of levels of stiffness stimuli, and some were conducted with materials that differed greatly in their composition and surface properties. Therefore, no existing studies have investigated the ability of human fingers to perceive stiffness using a wide range of stiffness levels in artificially controlled stimuli. Conversely, if researchers can create artificially presented stimuli whose stiffness can be tightly controlled, they can conduct experiments to investigate the human ability to perceive stiffness.

Based on the situation of stiffness perception research described above, the present study sought to develop a new material whose elasticity can be precisely controlled. Doi et al. [17] established a test-piece molding method incorporating paraffin oil and thermoplastic resins of the copolymer rubbers isoprene and butadiene. This allowed the development of new materials that could be precisely adjusted for a wide range of stiffness levels. In another study, Chiba et al. [18] used the same materials to clarify the precise degree of stiffness that university students perceived when they depressed these materials with their fingertips. Subsequent studies have clarified the relationship between finger-pad deformation and stiffness perception [19] and the relationship between the finger used to touch an object and stiffness perception [20]. Based on the results of these studies, educators involved in the training of acupressure massage therapy for the visually impaired have asked us to develop assessment materials to ascertain the stiffness perception ability of beginning learners who wish to become acupressure massage therapists.

Licensed acupressure massage therapy is one way for visually impaired individuals to have economic independence [21]. Licensed acupressure massage therapists perform palpations by pressing their fingers onto the body’s surface to detect tissue that is harder than the surrounding tissue [22]. Training courses for acupressure massage therapists are offered in Japanese schools for the blind, where visually impaired teachers instruct adult visually impaired students. It takes considerable time for novices to develop a personal sense of stiffness via palpation. This is because visual information cannot be used, and palpation skills are difficult to teach owing to subjective sensation. There is a demand among novice visually impaired acupressure masseurs for more quantitative and technical guidance and evaluation in palpation and other techniques [23]. In particular, a visually impaired licensed therapist needs to be able to perceive stiffness through their fingertips. Therefore, teachers endeavor to provide careful introductory education based on a quantitative assessment of new students’ basic stiffness perception. Although research has progressed toward the realization of quantitative education in acupuncture needle insertion [24], assessment materials to help teachers understand new students’ basic stiffness perception are lacking. Creating stimuli that can allow researchers to physically control the stiffness degree to examine the stiffness perception in detail is challenging. If we can develop evaluation materials for stiffness perception and clarify the differences in stiffness perception between teachers and beginning learners, teachers can also train beginning learners in stiffness perception.

Given that such perception is crucial to acupressure massage therapists [25], there is a demand for the development of educational assistive tools as an alternative to visual information for visually impaired teachers and the creation of assessment materials to clarify tactile ability quantitively. In this study, we developed suitable fundamental assessment materials that visually impaired licensed teachers could use to quantitatively assess the difference in stiffness perception ability in beginning learners and easily instruct them in the early stages of learning. We used these materials to compare the beginning learners’ ability to perceive stiffness with that of teachers.

## 2. Materials and Methods

### 2.1. Experimental Approach to the Problem

In this study, we developed assessment materials for stiffness perception to compare the beginning learners’ ability to perceive stiffness with that of teachers. The participants were recruited through research collaborators (teachers at schools for the blind) involved in the training of acupressure massage practitioners. The experiment took place in one of the schools for the blind in Japan. The recruitment period was from October to December 2018. Participation in the experiment was voluntary. Personal information about the participants was kept strictly confidential. Before starting the experiment, we provided the participants with an oral description of the experiment and obtained their informed consent. This experiment was conducted with the approval of Japan’s National Institute of Special Needs Education, Ethics Review for Research.

### 2.2. Participants

In this experiment, teachers from a school for the blind with more than 10 years of experience were asked to participate as experienced qualified licensed massage therapy teachers with visual impairments. The participants included five experienced qualified licensed massage therapy teachers with visual impairments (average age: 41.2 ± 6.2 years; mean experience: 20.0 ± 4.9 years; level of visual impairment: two males with blindness, one female, and two low vision males with a corrected vision of 20/66 or less). Based on the participants’ requests, the detailed medical histories of the visually impaired participants were not described, as this constitutes personal information; however, all participants had been visually impaired since school age. When selecting participants as beginning learners, we considered the absence of any special history of damages to the sense of touch. Based on the advice of qualified teachers from schools for the blind, the recruitment condition for the beginning learners was that they were sighted and had no prior experience with massage therapy to eliminate any effect of massage therapy experience on stiffness perception. We recruited five sighted persons (average age: 43.2 ± 13.9 years) who had no previous massage therapy experience to participate in this experiment as beginning learner candidates. As the number of adult students with acquired visual impairment enrolled in Japanese schools for the blind is increasing, we also requested the participation of this group. Although we wanted to increase the number of visually impaired teachers participating in the experiment, the number of participants was limited to five in consideration of the workload and busy schedule of each teacher. In addition, the number of novice participants matched the number of teacher participants. The small sample size is appropriate considering that this is a pilot study. We classified the teachers and beginning learners in this experiment as “qualified” and “unqualified” participants, respectively.

None of the participants had injuries or abnormalities in their superior limbs or their fingertips, and none had any impairment or associated medical history that could hinder their performance of the experimental tasks. All participants performed the experiment blindfolded. They were presented with successive pairs of eight different stimuli and were asked to report the difference in stiffness for each pair.

### 2.3. Assessment Materials

Acupressure massage therapists detect abnormally hard tissue in patients’ bodies through palpation. As mentioned earlier, the effective assessment of beginning learners’ stiffness perception necessitates the production of assessment materials whose stiffness can be precisely measured. Therefore, as a teaching aid for the initial instruction about stiffness perception, we produced assessment materials using thermoplastic elastomer materials that allow for incremental adjustments in elastic stiffness, following a previous study [17].

As for the motion during palpation, the pressure on the volume strain of an object with viscoelastic properties changes with the rate of compression [26]. It has been found that when practitioners perceive subtle differences in the stiffness of an object during palpation, they press their fingers into it at a certain speed [27]. Therefore, to reduce the effect of the pressing speed on stiffness perception, we decided to use elastic test pieces as assessment materials. The use of these materials enables one to grasp the difference in the basic stiffness perception ability between qualified and unqualified participants.

**Figure 1:**
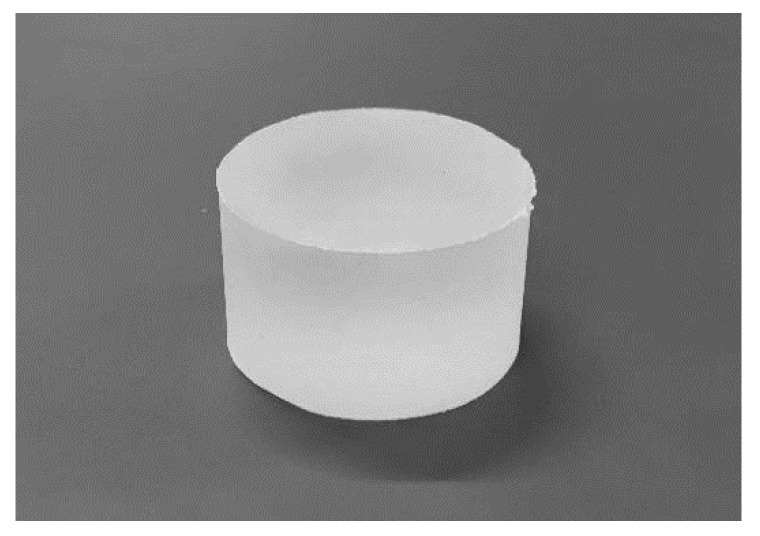
Test piece created from thermoplastic resin and paraffin oil.

An example of the assessment materials is shown in Figure 1. The materials were produced from a compound consisting of thermoplastic resin and paraffin oil. The thermoplastic resin is a block copolymer based on a polystyrene block and an elastomer block with a flexible polyolefin structure. The stiffness of the assessment material depends on the mixing ratio of the thermoplastic resin and paraffin oil; thus, it was possible to prepare materials with a wide range of stiffness levels. We poured the compound into a flat aluminum mold and formed it into a cylindrical shape 50 mm in diameter and 30 mm tall. Objects of this size are used by the Japanese Society of Rubber Science and Technology [28] to measure the stiffness of materials. We measured the reaction force using a compression-testing machine. To measure the stiffness of these materials, Young’s modulus [N/m^2^] was adopted, which is calculated on the basis of the reaction force (N) when a test piece is compressed to a certain ratio (in this study, 2/3 compression). We calculated Young’s modulus by following the methods of the Japanese Society of Rubber Science and Technology [28].

Chiba et al. [18] tested the relationship between the quantified stiffness of the thermoplastic resin materials and experiment participants’ perceptions of stiffness. To measure stiffness perception, the authors used a seven-stage scale in which participants described the stiffness of material as “extremely soft,” “fairly soft,” “slightly soft,” “neither,” “slightly hard,” “fairly hard,” or “extremely hard.” We developed our materials after accounting for the relationship between Young’s modulus and stiffness perception measured by Chiba et al. [18].

Eight experimental test pieces with a wide range of stiffness levels were created for this study (Table 1). The stiffness of these pieces corresponded roughly to the seven categories of stiffness sensation used by Chiba et al. [18]. Although stimuli E and F are in the same category of stiffness, their Young’s moduli values are different. We decided to adopt both stimuli E and F based on the advice of our collaborators, who suggested that we should determine whether experimental participants could distinguish between them precisely because they belonged to the same category of stiffness sensation.

### 2.4. Equipment

**Figure 2:**
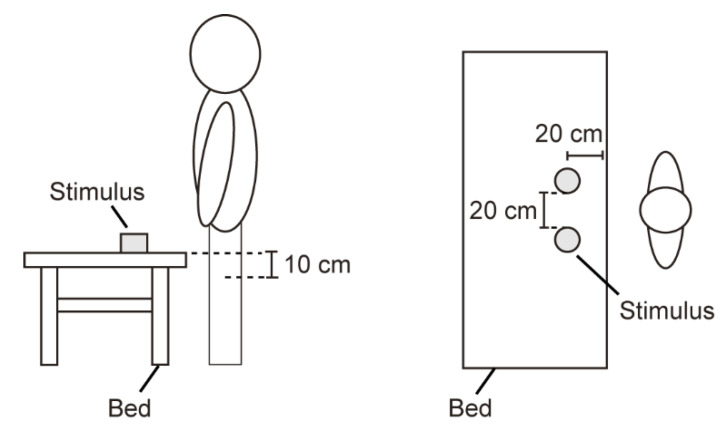
Location of the presented stimuli.

The test pieces were placed on an elevating bed (Roller Max, Electric Hydraulic Elevating Bed AK-1) to simulate the palpation of a patient. Pairs of stimuli were placed 10 cm above the base of the participants’ patella, 20 cm from the edge of the bed, and 20 cm from each other (Figure 2).

### 2.5. Conditions

Only stimuli B, C, D, E, F, and G were used as standard stimuli in the experiment. They were presented to participants in pairs of stimuli from adjacent categories (i.e., the next softest and next hardest stimuli), along with the comparative stimulus from the same category (i.e., a matching stimulus). This is described below in more detail. We could have produced materials that were softer than stimulus A (the softest) or harder than stimulus H (the hardest); however, these materials would not retain their cylindrical shape. Therefore, stimuli A and H were not used as standard stimuli.

### 2.6. Procedure

**Figure 3:**
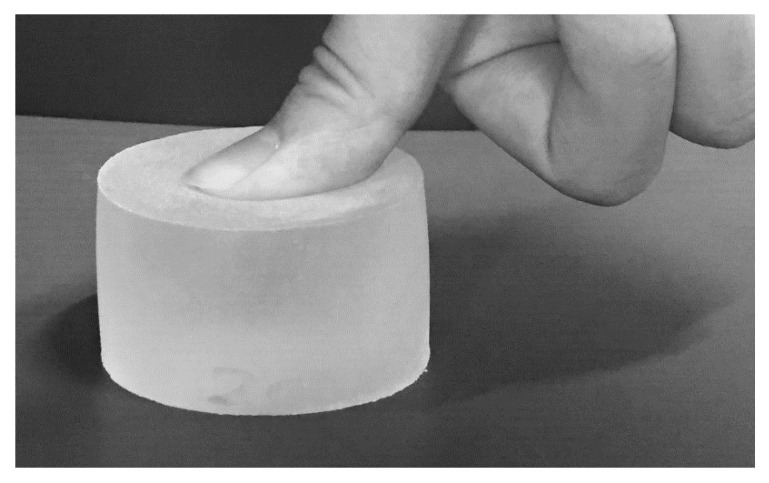
Example of depressing the centers of one test piece.

**Figure 4:**
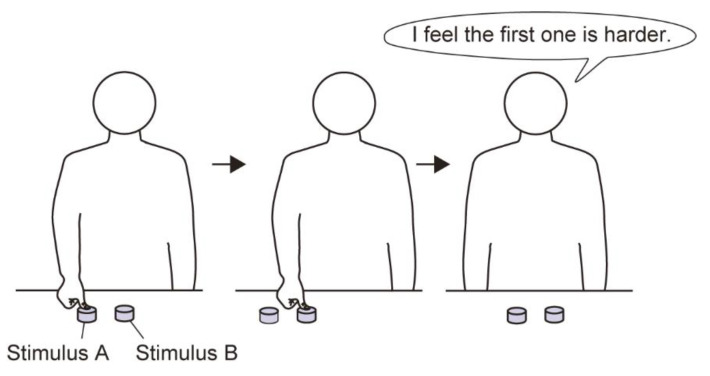
Example of the experimental procedure (discrimination task using stimuli A and B).

The participants examined two stimuli in each trial; they were asked to depress the centers of these stimuli once for each trial with their thumb for no more than three seconds each and at time intervals of no more than three seconds (Figure 3). We asked participants to use their right thumb to ensure consistency among participants, based on the advice of our research collaborators. Participants were also blindfolded to eliminate any effect of various visual information and to control the experimental conditions between participant groups. After depressing both stimuli, they reported whether they felt that one or the other was hard or soft, or whether they felt that both were the same. They were also asked to report their level of certainty regarding their judgment of the materials’ stiffness on a five-point scale (1 = not certain; 5 = certain). Participants undertook sufficient practice trials to familiarize themselves with the experiment. An example of the experimental procedure is shown in Figure 4.

For each standard stimulus (B, C, D, E, F, G), we compared three stimuli: one each from the stiffness categories on either side of the standard stimulus and one from the same category of stiffness. For example, for the standard stimulus B, we tested the following five combinations of stimuli pairs: A–B and B–A (preceding category), B–B (same category), and B–C and C–B (succeeding category). Of these, A–B, B–A, B–C, and C–B were presented twice, and B–B was presented four times. Accordingly, we conducted 12 trials for the six standard stimuli. The total experiment time was approximately one hour. We counterbalanced the order in which stimuli were presented to eliminate the effect of the order. Our experiment was conducted at almost the same time of the day on different days for all participants.

### 2.7. Evaluation Indices

The correct answer rate was adopted as the index to objectively evaluate participants’ stiffness perception. A subjective evaluation index was also adopted; participants self-assessed their confidence through their answers on the aforementioned five-point scale. The mean values of the percentage of correct answers and levels of certainty were calculated for each standard stimulus for each subject. Each of these mean values was analyzed according to the statistical analysis method described in Section 2.8.

### 2.8. Analytical Method

After collecting the participants’ correct answer rate and levels of certainty, we calculated the average correct answer rate and level of certainty for each of the six standard stimuli. The normality of the variable distribution was checked with the Shapiro–Wilk test, and the Levene test was used to evaluate variance homogeneity. If data meet the assumption of normality and variance homogeneity, we conducted a *t*-test with a significance level of 5% to confirm the differences between the teachers’ and beginning learners’ correct answer rate and level of certainty. We used Cohen’s *d* values and *r* values to evaluate their correct answer rate and certainty level. When the normality of the variable distribution was not observed, Mann–Whitney’s *U* test was conducted. All analyses were performed in IBM SPSS Statistics V23 with the level of significance set at 5%.

## 3. Results

**Figure 5:**
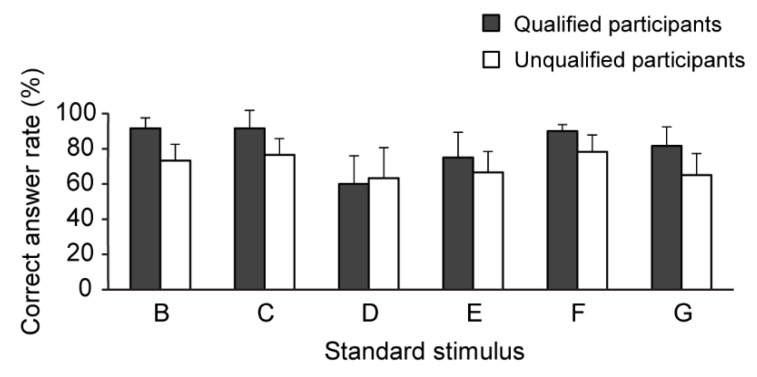
Correct answer rates for each standard stimulus.

**Figure 6:**
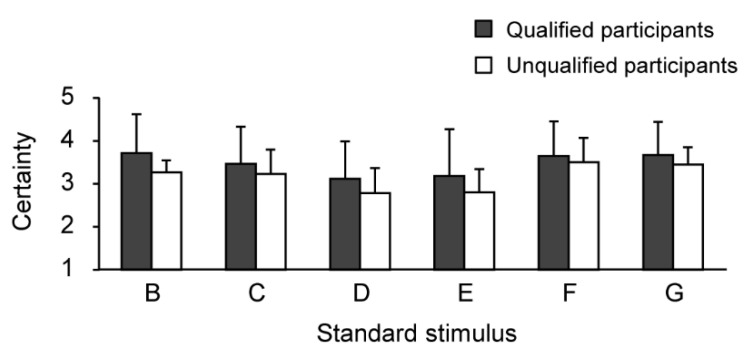
Levels of certainty for each standard stimulus.

Figure 5 and Figure 6 and Table 2, Table 3 and Table 4 show the results concerning the correct answer rates and levels of certainty for each standard stimulus. For the standard stimulus B, there was a significant difference between qualified and unqualified participants’ correct answer rates: *t*(8) = 3.77, *p* < 0.01, *d* = 2.39. In contrast, there was no significant difference between qualified and unqualified participants’ certainty levels: *t*(8) = 1.06, *p* = 0.32, *d* = 0.67.

Regarding the standard stimulus C, there was a significant difference between qualified and unqualified participants’ correct answer rates: *U* = 3.00, *p* < 0.05, *r =* 0.64. In contrast, there was no significant difference between qualified and unqualified participants’ certainty levels: *t*(8) = 0.50, *p* = 0.63. *d* = 0.32.

For the standard stimulus D, there was no significant difference between qualified and unqualified participants’ correct answer rates (*U* = 9.50, *p* = 0.51, *r* = 0.21) and between qualified and unqualified participants’ certainty levels (*t*(8) = 0.71, *p* = 0.50, *d* = 0.45).

Regarding the standard stimulus E, there was no significant difference between qualified and unqualified participants’ correct answer rates (*U* = 7.50, *p* = 0.28, *r* = 0.34) and between qualified and unqualified participants’ certainty levels (*t*(8) = 0.70, *p* = 0.50, *d* = 0.44).

As for the standard stimulus F, there was a significant difference between qualified and unqualified participants’ correct answer rates: *U* = 3.50, *p* < 0.05, *r* = 0.64. In contrast, there was no significant difference between qualified and unqualified participants’ certainty levels: *t*(8) = 0.34, *p* = 0.74, *d* = 0.22.

In regard to the standard stimulus G, there was no significant difference between qualified and unqualified participants’ correct answer rates (*t*(8) = 2.27, *p* = 0.05, *d* = 1.43) and between qualified and unqualified participants’ certainty levels (*t*(8) = 0.55, *p* = 0.60, *d* = 0.35).

**Figure 7:**
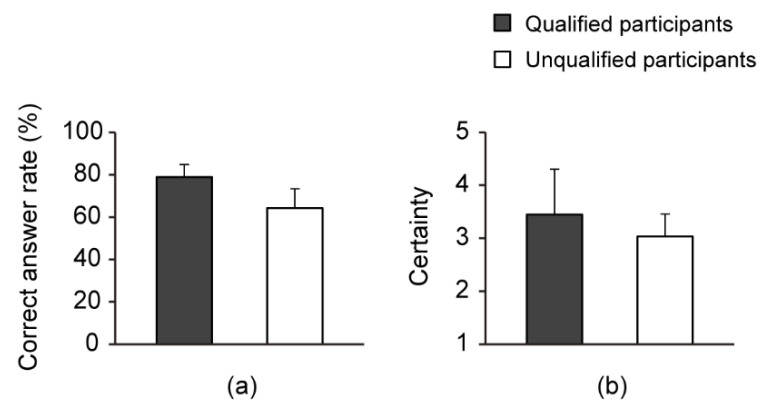
Correct answer rates (**a**) and levels of certainty (**b**).

Finally, Figure 7 and Table 5 show the average correct answer rate and level of certainty for all standard stimuli. This shows that there was a significant difference between qualified and unqualified participants’ correct answer rates: *t*(8) = 3.25, *p* < 0.05, *d* = 2.05. In contrast, there was no significant difference between qualified and unqualified participants’ certainty levels: *t*(8) = 0.97, *p* = 0.36, *d* = 0.62.

## 4. Discussion

We used our developed assessment materials to compare the beginning learners’ ability to perceive stiffness with that of the teachers. Compared to beginning learners, teachers answered correctly at an overall significantly higher rate. These results revealed areas of stiffness that are difficult for beginning learners to identify.

### 4.1. Differences between Qualified and Unqualified Participants’ Perception of Stiffness

Qualified participants showed a significantly higher correct answer rate for stimuli B, C, and F than their unqualified counterparts. The effect size of these results was high, indicating a remarkable difference between the two groups. At the significance level used in this study, there was no statistically significant difference between qualified and unqualified participants in the correct answer rate for stimulus G. However, the high effect size suggests that qualified participants seemed to have higher stiffness perception ability for stimuli G compared to unqualified counterparts. These results might be attributed to the fact that, as qualified massage therapy teachers, the qualified participants engage in palpation and treatment almost daily and thus are more experienced than their unqualified counterparts. Previous research [29] and our professional research collaborators suggested that therapists’ knowledge of anatomy, their patients’ skin temperature, and irregularities in their patients’ subcutaneous tissue helped them perceive stiffness as well. Although this experiment is limited to stiffness perception, the quantitative knowledge that such a large gap exists between qualified and unqualified participants’ perception of stiffness is useful for teachers to examine their teaching methods. The use of our assessment materials in the field of education might close this relatively large gap. To claim that the gap in stiffness perception between the groups can be reduced on the basis of the results of this experiment, it will be necessary to confirm the change in stiffness perception with the continued use of our assessment materials in the future.

In contrast, no significant differences were found between qualified and unqualified participants’ correct answer rates for standard stimuli D and E. According to Maeno et al. [30], Young’s modulus of human subcutaneous tissue is 3.40 × 10^4^ (N/m^2^), or 4.53 when converted into logarithmic form. Meanwhile, Young’s moduli for standard stimuli D and E in this experiment were 4.50 and 4.68, respectively. Considering that it might be difficult for participants to perceive the stiffness of an object if the object is of the same or similar stiffness to human fingertips, it might be difficult for even experienced massage therapists to discern the stiffness of stimuli D and E with certainty. In addition, these stimuli were categorized more or less in the middle of Chiba et al.’s [18] seven-point scale of stiffness sensation; thus, it may be difficult for any participant, qualified or not, to perceive significant differences between these stimuli through palpation.

There were no significant differences in levels of certainty between participants in any of the standard stimuli. In this experiment, the qualified participants came into contact for the first time with our experimental stimulus, which was different from human skin, and thus, they had to determine the hardness with the same level of certainty of the unqualified participants.

As expected, we observed no significant difference between qualified and unqualified participants’ correct answer rates and levels of certainty for standard stimuli D and E. These results indicated that when participants’ correct answer rate was low, their certainty tended to be lower as well. The participants only touched the presented stimuli once in this experiment, which may have affected the results. If our qualified participants palpated human skin through their usual methods (i.e., for an extended period), we might see more significant differences between qualified and unqualified participants’ perception of human skin similar to the stiffness of stimuli D and E.

Although we need to improve this assessment material, as a first step, we could develop suitable fundamental assessment materials that visually impaired licensed teachers could use to quantitatively assess the difference in the stiffness perception ability of beginning learners and easily educate them in the early stages of learning. One advantage of these educational assistive tools that provide an alternative to visual information is that visually impaired teachers can intuitively see the combination of presented stimuli that often poses difficulties for students in perceiving stiffness.

Our experiment was conducted at almost the same time of the day on different days for all participants. Since circadian changes affect pain perception [31], the possibility of their effect on stiffness perception should also be considered. It would be worthwhile to conduct an experiment to investigate the relationship between circadian changes and stiffness perception.

### 4.2. Limitations and Future Research Directions

Future studies need to delve into these assessment materials and stiffness levels in more detail to consider adequate learning methods for beginning learners. As human skin has a viscoelastic nature, unlike the elastic material used in this experiment [32], assessment materials with viscoelastic properties will be required for the advanced stages of education. If the stimulus presented in this study had been more similar to human skin, we might have observed greater differences between qualified and unqualified participants’ perception of stiffness. Future experiments should test viscoelastic stimuli that are closer in texture to human skin. Regarding practical interventions in the occupational training of visually impaired people, this study has a few limitations. For instance, to eliminate any effect of massage therapy experience on stiffness perception, our study participants were sighted persons rather than visually impaired students with total blindness or low vision. Thus, additional considerations are necessary when gauging how these assessment materials might be used by blind students. For example, in a future study focused on helping visually impaired students learn to perceive stiffness, a guidebook with large printed visual characters and additional features, such as Braille and/or audio instructions, should accompany these materials during training.

### 4.3. Implications for Professionals

First, although this study focused on stiffness perception in the early stages of learning, it enabled a quantitative evaluation of stiffness perception. Second, it produced a unique and innovative assessment material. This is significant because it is not easy in practice to control the properties of viscoelastic materials with high precision, let alone produce similar stimuli with different levels of stiffness. Third, this study was an active attempt to carry out practical experiments in cooperation with a school for the blind to provide vocational training for the visually impaired.

There are admittedly many improvements to be made in the practical application of the materials to assess the stiffness perception of visually impaired beginning learners of acupressure massage. In particular, there is a need to promote practical research using materials closer to human skin, rather than focusing on hardness identification in the early stages of learning.

## 5. Conclusions

This study aimed to develop suitable fundamental assessment materials that could be used by visually impaired licensed teachers to quantitatively assess the differences in the stiffness perception ability of beginning learners in the early stages of learning. We used these materials to compare the beginning learners’ ability to perceive stiffness with that of the teachers. Compared to beginning learners, teachers answered correctly at an overall significantly higher rate. Specifically, the teachers’ correct response rate (78.8%) for the stiffness perception of all presented stimuli was approximately 15% higher than the beginning learners’ correct response rate (64.2%). These results revealed areas of stiffness that are difficult to identify for beginning learners. In the future, we hope that these assessment materials will help beginning learners to train their perception of stiffness. Furthermore, these materials might assist and motivate beginning learners to understand their ability to perceive stiffness, given their need for nonvisual study materials.

## Figures and Tables

**Table 1:** Stiffness of the test pieces used in the experiment.

Stimulus	Young’s Modulus (log)	Categories
A	3.79	extremely soft
B	4.16	fairly soft
C	4.33	slightly soft
D	4.50	neither
E	4.68	slightly hard
F	4.89	slightly hard
G	5.26	fairly hard
H	5.55	extremely hard

**Table 2:** Results of teachers’ and beginning learners’ correct answer rate.

Standard Stimulus	Mean (SD)		Median (Min/Max)	
	Qualified Participants	Unqualified Participants	Qualified Participants	Unqualified Participants
B	91.7 (5.3)	73.3 (8.2)	91.7 (83.3/100)	75.0 (58.3/83.3)
C	91.7 (9.1)	76.7 (8.2)	91.7 (75.0/100)	83.3 (66.7/83.3)
D	60.0 (14.3)	63.3 (15.5)	66.7 (33.3/75.0)	66.7 (33.3/75.0)
E	75.0 (13.0)	66.7 (10.5)	66.7 (66.7/100)	58.3 (58.3/83.3)
F	90.0 (3.3)	78.3 (8.5)	91.7 (83.3/91.7)	75.0 (66.7/91.7)
G	81.7 (9.7)	65.0 (13.3)	83.3 (66.7/91.7)	58.3 (50.0/83.3)

**Table 3:** *t*-test and Mann–Whitney *U* test with a significance level of 5% to confirm the differences between teachers’ and beginning learners’ correct answer rate.

Standard Stimulus	*p*-Value Significance Level (Effect Size)
B	0.005 (2.39)
C	0.043 (0.64)
D	0.511 (0.21)
E	0.283 (0.34)
F	0.043 (0.64)
G	0.053 (1.43)

**Table 4:** T-test with a significance level of 5% to confirm the differences between teachers’ and beginning learners’ certainty level.

Standard Stimulus	Mean (SD)		*p*-Value Significance Level (Effect Size)
	Qualified Participants	Unqualified Participants	
B	3.7 (0.8)	3.3 (0.2)	0.319 (0.67)
C	3.5 (0.8)	3.2 (0.5)	0.627 (0.32)
D	3.1 (0.8)	2.8 (0.5)	0.499 (0.45)
E	3.2 (1.0)	2.8 (0.5)	0.502 (0.44)
F	3.7 (0.7)	3.5 (0.5)	0.741 (0.22)
G	3.7 (0.7)	3.5 (0.4)	0.595 (0.35)

**Table 5:** *t*-test with a significance level of 5% to confirm the differences between teachers’ and beginning learners’ correct answer rate and certainty level.

Evaluation Indices	Mean (SD)		*p*-Value Significance Level (Effect Size)
	Qualified Participants	Unqualified Participants	
correct answer rate	78.8 (5.3)	64.2 (7.3)	0.011 (2.05)
certainty level	3.5 (0.8)	3.0 (0.4)	0.358 (0.62)

## Data Availability

The data that support the findings of this research are available from the corresponding author on reasonable request. The data are not publicly available because they contain information that could compromise the privacy of research participants.
